# Post-Weld Heat Treatment of API 5L X70 High Strength Low Alloy Steel Welds

**DOI:** 10.3390/ma13245801

**Published:** 2020-12-18

**Authors:** Houman Alipooramirabad, Anna Paradowska, Shahrooz Nafisi, Mark Reid, Reza Ghomashchi

**Affiliations:** 1 School of Engineering, University of British Columbia, Okanangan, BC V1V 1V7, Canada; 2 School of Mechanical Engineering, the University of Adelaide, Adelaide, SA 5005, Australia; shahrooznafisi@gmail.com (S.N.); reza.ghomashchi@adelaide.edu.au (R.G.); 3 Australian Nuclear Science and Technology Organisation (ANSTO), Lucas Heights, NSW 2234, Australia; anp@ansto.gov.au (A.P.); markr@ansto.gov.au (M.R.); 4 School of Civil Engineering, The University of Sydney, Sydney, NSW 2006, Australia

**Keywords:** PWHT, neutron diffraction, HSLA, microstructural characterization, EBSD

## Abstract

High Strength Low Alloy (HSLA) steels are the materials of choice in pipeline construction with the API X70 grade as the steel for the majority of pipeline networks constructed during the late 20th and early this century. This paper reports on the influence of Post-Weld Heat Treatment (PWHT) on the reduction of residual stresses, resulting changes in the microstructure, and mechanical properties of a multi-pass, X70 HSLA steel, weld joints made by a combined Modified Short Arc Welding (MSAW) and Flux Cored Arc Welding (FCAW) processes. Neutron diffraction results highlighted high magnitude of tensile residual stresses, in excess of yield strength of both parent and weld metal, in the as-welded specimen (~650 MPa), which were decreased substantially as a result of applying PWHT (~144 MPa). Detailed microstructural studies are reported to confirm the phase transformation during PWHT and its interrelationship with mechanical properties. Transmission Electron Microscopy (TEM) analysis showed polygonization and formation of sub-grains in the PWHT specimen which justifies the reduction of residual stress in the heat-treated weld joints. Furthermore, microstructural changes due to PWHT justify the improvement in ductility (increase in the elongations) with a slight reduction in yield and tensile strength for the PWHT weld joint.

## 1. Introduction

Amongst a range of welding techniques employed in manufacturing complex structures, the combination of MSAW and FCAW processes are claimed to render a more economical advantage through reduction in welding time and less demand on welder skill [1]. This is because of low production rate and demand for highly skilled welder for the more customary practice of Shielded Metal Arc Welding (SMAW) [2,3]. MSAW and FCAW are two distinct processes, although they have many similarities in equipment and application. Both processes utilize a continuous wire-feed as the filler metal, and both employ gas and a flux to shield the arc and Weld Metal (WM) as well as preventing the molten metal from oxidation [4,5].

Both processes can be implemented in semi or fully automated mode and thus should have cost advantages over the other commonly used processes like SMAW [1,6]. Study conducted by Alipooramirabad et al. [7] has shown that using combined MSAW and FCAW processes improves weld joint integrity and enabling high deposition rate while reduces distortion along with better adaptability and ease of equipment use. Moreover, FCAW can provide better control over the applied weld current and heat input leading to a more uniform weld composition, less dilution, and smaller grain size; all enhancing the mechanical properties and contributing to service performance of the welded structures [8,9,10,11]. The MSAW brings about low-spatter droplet transfer, high process stability, and the ability to deposit weldments with greater control of the heat input [12]. Despite such technological advantages, high level of tensile residual stresses, greater than SMAW, were found to be generated during the multi-pass welding with combined MSAW and FCAW for HSLA steel welds [6,7,13]. The tensile residual stresses are particularly detrimental to the service life of the welded structures and can increase the risk of fatigue, environmentally assisted cracking (Hydrogen Induced Cracking-HIC and Stress Corrosion Cracking-SCC) and fracture in the welded joints. Such detrimental effects were confirmed in the study conducted by Cheng et al. [14], where tensile residual stress decreased the crack threshold value and fatigue resistance of high strength steel welds. Similar observations were also made in the studies conducted by Katsuyama et al. [15] on the SCC growth in 316 L stainless steel as well as Chang and Lee [16] on the increase of the *J*-integral values (i.e., increase crack propagation) in high strength steels in the presence of tensile residual stresses. Stress-relieving such as PWHT is therefore mandatory, mainly to minimize the risk of brittle fracture. PWHT can lead to a substantial reduction of tensile residual stresses in the welded joints [17] and, to some extent, tempering of the Heat Affected Zone (HAZ) and the WM microstructures [18,19,20]. PWHT of C-Mn steels such as HSLA steels is generally performed at approximately 600 °C, for about one hour for every 25 mm of thickness [21,22,23]. 

There is a significant amount of work published on the PWHT process parameters and effects on the residual stress [24,25,26], microstructure [27,28] and mechanical properties [29,30] of the treated welded joints. For example, a study conducted by Paddea et al. [25] for P91 steel pipe girth welding shows a substantial reduction of tensile residual stresses after PWHT, while Zhang et al. [31] identified microstructural changes (i.e., reduction of dislocation and precipitation of carbonitride) during PWHT, which can have a significant impact on the tensile properties of individual microstructures. Study conducted by Araújo et al. [27] for grade X70Q showed that normalizing heat treatment at 920 °C promoted formation of a ferrite/pearlite structure which lead to reduction in the ultimate tensile strength of the joints equivalent to grade X46 steel (below tensile strength of grade X70Q), while heat treatment at 600 °C proved to be beneficial for the impact toughness in the HAZ and the Fusion Zone (FZ) and still maintained the tensile strength of X70Q grade steel, mainly due to the reduction of the volume fraction of the Martensite–Austenite (M–A) and Martensite–Austenite–Bainite (MAB) constituents. Similar observation was made by Filho et al. [29] where applying normalizing heat treatment for low alloy steel weld metals led to microstructural changes (fine grained microstructure was changed to a coarse equiaxed ferrite with ferrite-carbide aggregates) and significant reduction in the yield and tensile strength of the WM. 

Mitra, et al. [24] conducted experimental investigation (x-ray diffraction) with numerical validation on the effects of time and temperature of PWHT on stress relief of very thick (800 mm thickness) ferritic steel with narrow-gap weldment. The residual stress was found to significantly decrease during the initial stages of holding time while the rate of decreasing residual stresses was significantly lower for the later stages of the holding time. Olabi and Hashmi [26] investigated the effects of PWHT on the residual stresses and mechanical properties in welded structural steel. They have reported that PWHT leads to a significant reduction of the residual stresses, ~70%, as well as some improvement of the toughness by about 15%. Paddea, et al. [25] used neutron diffraction method to investigate the effects of PWHT on the distribution of residual stresses in a P91 (8CrMoV–Mn) steel-pipe girth weld. A high level of tensile residual stresses was observed in the as-welded condition with the maximum of 600 MPa, while after PWHT residual stresses were reduced to 120 MPa (~24% of yield strength of the parent metal-PM). Ravi, et al. [32] utilized a linear elastic fracture mechanics approach to investigate the effects of PWHT on the fatigue life and fatigue crack growth behavior of the mis-matched HSLA steel welds (undermatched, matched, and over matched). It was found that PWHT has minor impact on reducing the strength and improving the impact toughness of WM, but it leads to a decrease in the hardness and an increase in the percentage of elongation. Furthermore, the fatigue performance for all of the mismatched welded joints were improved after PWHT, which could be due to the reduction in the magnitude of tensile residual stresses in the weld region and the vicinity. Ramkumar et al. [28] investigated the effects of PWHT on the microstructure and mechanical properties of activated flux TIG welds of Inconel 750. In contrast to the above-mentioned studies, it was confirmed that PWHT leads to improvements in tensile properties of weldments (the tensile strength for the as-welded and after PWHT coupons were 736 and 1142 MPa, respectively). Moreover, the joint efficiencies ((UTS_Weld_/UTS_Parent metal_) × 100) of the as-welded and PWHT weldments were found to be 60.7% and 94.07%, respectively. On the other hand, the impact toughness of the as-welded sample was found to be higher than the PWHT coupons, which was attributed to the lower hardness experienced in the fusion zone in the as-welded specimen (higher hardness in the PWHT coupons was associated with the formation of smaller grain size and the presence of M_23_C_6_ carbides at the grain boundaries of the Inconel X750 welds). Zhang, et al. [31] utilized microstructure-based finite element analysis to investigate the effect of PWHT on the mechanical properties of individual microconstituents of C–Mn WM. It was found that PWHT leads to significant changes into the strength of WM by altering the strength of individual microconstituents. They reported softening of the grain boundary ferrite irrespective of the holding temperature or softening of acicular ferrite after heat treating at 400 °C and 700 °C. The softening and the changes into the tensile properties of the individual microconstituents were associated with the reduction of dislocation density and precipitation of carbonitride. Such strength variation between acicular ferrite and grain boundary ferrite after the PWHT leads to the plastic strain localization of the WM during tension and the critical fracture strain of WM.

In spite of a large body of work on the effects of PWHT operation on the microstructure and mechanical properties of a wide range of HSLA steels and welding processes combination, to the best of authors’ knowledge, there are limited studies on the PWHT of combined MSAW-FCAW multi-pass welding of X70 HSLA steel so far. The X70 steel is widely used in the energy distribution and transportation sectors, and therefore, the integrity of weld joint is of utmost importance as they are exposed to environmental assisted cracking, e.g., HIC [33]. The current study, as part of a major study on the welding of X70 line pipe steel, was therefore carried out to firstly explore the resulted residual stresses generated for this MSAW-FCAW combined welding route and then characterize in detail the effect of the applied PWHT procedure on the changes in the residual strains/stresses and the microstructural and mechanical properties of X70 HSLA multi-pass welded joints.

## 2. Experimental Procedure

### 2.1. Materials and Sample Preparation

**Figure 1:**
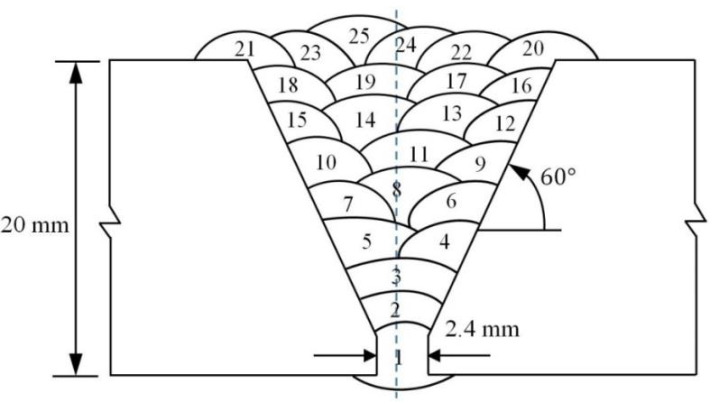
Schematic of the weld joint geometry and the weld deposition sequence in the welded specimens.

The test specimens utilized in this study were two steel plates (API 5L grade X70 [34]), with the thickness of 20 mm and the dimensions of 250 × 200 mm^2^. Figure 1 shows the preparatory joint geometry of the steel plates. Two samples were fabricated utilizing identical welding parameters and procedures. One was used to evaluate the residual stresses before and after PWHT and the other one used to prepare stress-free sample via electro-discharge machining (EDM) to relieve residual stresses from the WM and the HAZ. It is worth mentioning that the Charpy, tensile and hardness samples were also taken from both samples (as-welded and PWHT specimens) after neutron diffraction residual stress measurements. 

The root pass of the V-prep joint was completed with MSAW (pass 1-MSAW), and FCAW was used for the remaining passes (pass 2–25). Two different filler materials were used for welding of the specimens; ER70s-6 electrode and E81TNi class Flux cored wire were used to complete the root pass (pass 1) and the remaining passes (pass 2–25) of the welded joints, respectively. The sketch of the deposited weld is shown in Figure 1, and the chemical compositions of the consumable (electrodes) and base material are presented in Table 1 (for base material, measurement of nitrogen and carbon content were done by using ELTRA and LECO combustion analyzers, respectively).

### 2.2. Post-Weld Heat Treatment

**Figure 2:**
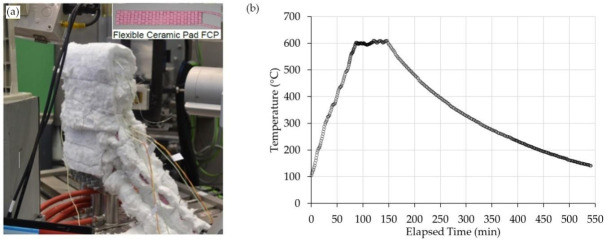
(**a**) Experimental set-up for Post-Weld Heat Treatment (PWHT), and (**b**) time-temperature graph during the PWHT cycle.

Heating blankets with block shaped elements which are made up of sintered alumina, containing resistance heating Ni-Cr wire, are used to carry out PWHT in this experimental investigation. The experimental set up is shown in Figure 2a including a picture of ceramic heating blanket. Advantage3 controller (temperature controller manufactured by Stork-Cooperheat, Sydney, Australia) was used to control heating blankets and to monitor the input current in accordance with a centrally mounted control thermocouple. Six thermocouples of K-type were attached (spot welded) to the specimen at various locations to monitor the temperature uniformity during heat treatment.

The heating setup and instrumentation allowed maintaining the set-temperature across the entire sample within an accuracy of ±5 °C variation. The as-welded sample was reheated at 5 °C/min to the soaking temperature of 600 °C and with the holding time of 60 min, as shown in Figure 2b. 

### 2.3. Mechanical Testing

The welded specimens were sectioned transversely to the welding direction (parallel to the rolling direction) and ground and mirror-like polished using 1 μm diamond paste for hardness evaluation of the PM, HAZ, and WM. Microhardness testing was performed according to the Australian standard AS.2205.6.1 [37], with the surface finishing as recommended by the standard as well as using light etching to define the HAZ/Weld zone and proper positioning of hardness indentations. Measurements were taken across the weld in the transverse direction (mid-plate thickness) and in the normal direction (through the thickness at the weld centerline) utilizing a Vickers (HV) indenter with the indentation force of 0.5 kg and a loading dwell time of 15 s. The distance between the hardness indentations was kept at 0.5 mm apart. 

**Figure 3:**
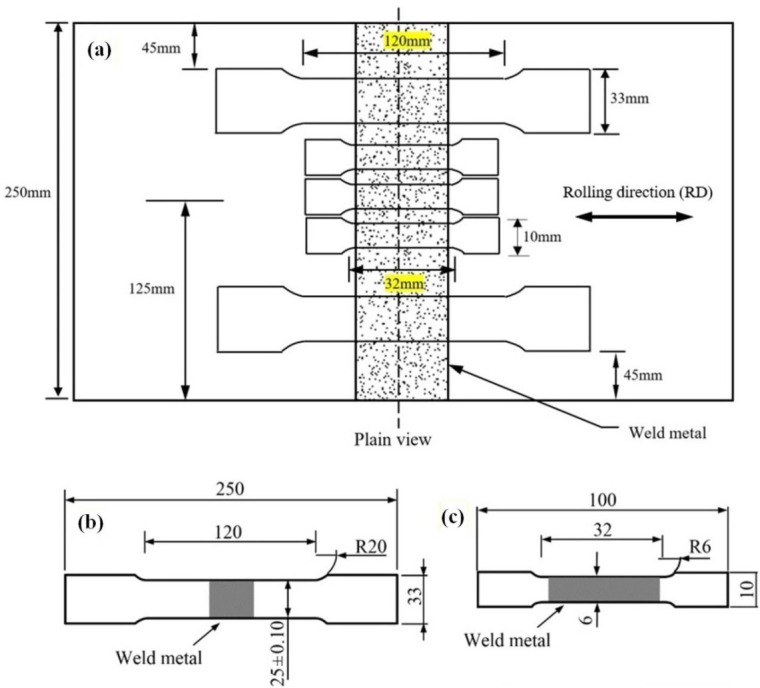
(**a**) Schematic representation of tensile test coupons prepared from the welded plates (yellow highlights show the gauge length of samples); (**b**) standard size tensile and (**c**) sub-sized tensile specimens (all dimensions are in mm).

Standard and sub-sized samples tensile testing were performed at room temperature with strain rate of $4\;\times \;{10\;}^{-4}s^{-1}\;$using two different universal tensile testing machines (INSTRON 1282 Servo-hydraulic and MTS with the loading capacity of 1000 kN and 300 kN, respectively). Both tensile specimens (standard and sub-sized specimens) were designed to fully characterize the mechanical properties of the PM and WM (note that the gauge length of the sub-sized specimens were fully covered the weld and HAZ). Tensile testing procedure and specimen specifications were conducted according to the ASTM-A370 and Australian standard AS.2205.2.1 [38,39]. The tensile tests were carried out to investigate the PWHT effects in both the PM and WM. The tensile test specimens were prepared in various locations transverse to the weld, as shown in Figure 3.

**Figure 4:**
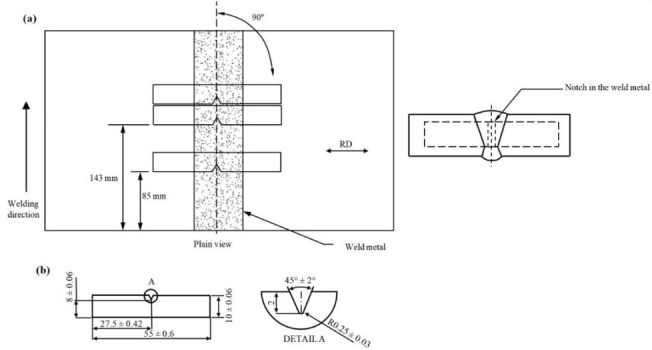
Schematic and dimensions of Charpy v-notch specimens for the impact test (**a**,**b**). (all dimensions are in mm).

Charpy impact tests were conducted at room temperature for the as-welded and PWHT specimens on standard size Charpy V-notch specimens (10 × 10 × 55 mm^3^). The locations within the welded plates and dimensions of the Charpy specimens are presented in Figure 4. The Charpy test was conducted in accordance with standard test methods for notch bar impact testing of metallic materials [40].

### 2.4. Microstructural Analysis

The macro- and micro-structural examinations were carried out for both the as-welded and PWHT specimens, sectioned perpendicular to the welding direction by keeping the weld as the center. Optical Microscopy (OM), Scanning Electron Microscopy (SEM), and Electron Backscatter Diffraction (EBSD) were used to examine the microstructure of the PM, the HAZ, and the Weld Metal (WM). Two different etchants were used for OM analysis including 5% Nital (5% nitric acid in 95% ethanol) as well as double etching procedure of 2% Nital (2% nitric acid in 98% ethanol) with 2% Picral (2% picric acid in ethanol). For EBSD analysis, sample polishing were conducted in two steps, initial polish with a semi-automatic TegraPol polishing machine [41], and the final step was completed by using a porous neoprene disc with a colloidal silica suspension (0.04 μm).

SEM (FEI Helios Nanolab 600) equipped with an EBSD detector (EDAX Hikari™) was utilized with the acceleration current and voltage of 2.7 nA and 30 kV of the electron beam, respectively. The step size was 30 nm with a hexagonal scan grid (scans were 100 × 100 μm^2^). TSL-OIM software (version 7) was used for the data collection and analyses in this study. TEM analysis was also conducted to fully characterize the microstructure of the as-welded and PWHT specimens. A 200kV TEM, Tecnai G2 Spirit, was employed and the samples (thin foils) with 15 μm × 10 μm × 75 nm dimensions were prepared using a dual beam Focused Ion Beam (FIB) scanning electron microscope (FEI Helios Nanolab 600). TEM samples were prepared from the PM, WM, and CGHAZ of the as-welded and post-weld heat treated specimens.

## 3. Results and Discussions

### 3.1. Residual Stress/Strain Measurement Prior and After PWHT

Neutron diffraction residual strain/stress measurements for the as-welded and PWHT specimens were conducted on KOWARI, the strain diffractometer at ANSTO (Sydney, Australia). A monochromatic neutron beam with the wavelength of 1.67 Å and gauge volume of $3\times 3\times 3{\;\mathrm{mm}}^{3}$ was used in the present investigation to measure the scattering angle from the α-Fe (211) reflection. Residual strains/stresses were measured at 76 points (38 points for each sample) in three principal directions. Electrical Discharge Machining (EDM), with a wire diameter of 0.2 mm, was used to prepare the stress-free samples and to obtain 6mm thick slices from across the weld ($6\times 80\times 20\;{\mathrm{mm}}^{3})$ at the centre of the plate. The stress-free or matchstick samples were cut from an area in such a way as to relieve all residual stresses (stress free sample is defined as a cut out of the plate with identical parameters similar to as-received plate to acts as the base line for strain calculation). The measurements of stress-free lattice spacing (d_0_) for the as-welded and PWHT specimens were conducted using the same measurement points as per corresponding interplanar spacing points with $3\times 3\times 3{\;\mathrm{mm}}^{3}$ gauge volume. Residual strain/stress measurements were conducted in three principal directions including longitudinal (parallel to the welding direction), normal (through thickness of the plate) and transverse (perpendicular to the weld) orientations. Further details of instrumentation and fundamentals of neutron diffraction can be found elsewhere [42,43,44,45]

**Figure 5:**
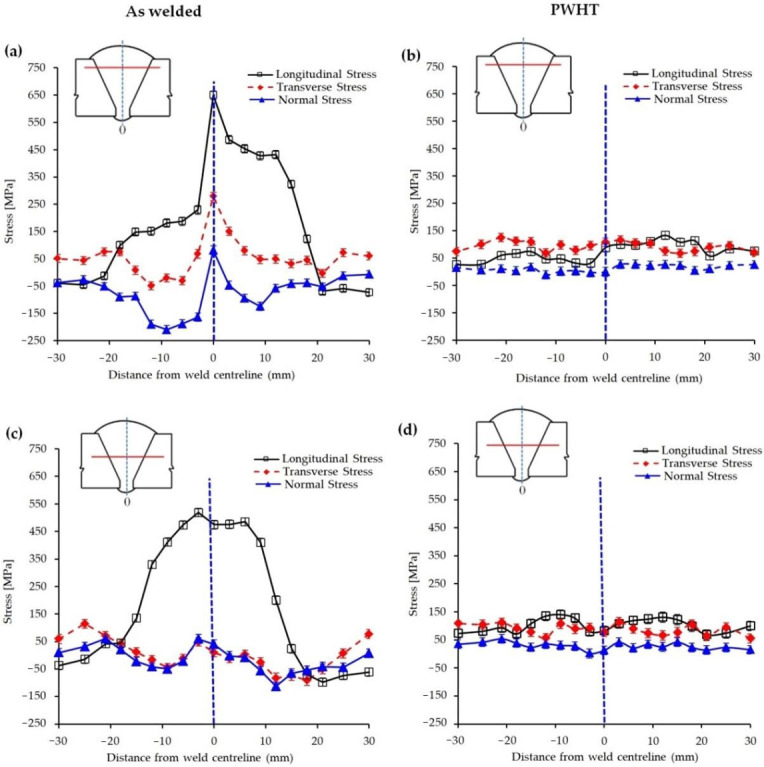
Residual stress distribution within 3 mm below the weld surface across the weld (**a**,**b**) and mid-thickness of the plate (**c**,**d**) for as–welded and post–weld heat treated specimens.

Figure 5a–d shows the distribution of residual stresses across the weld (3 mm below the weld top surface and mid-plate thickness) for the as-welded and PWHT specimens. Tensile residual stress higher than the yield strength of both the parent and weld metal (the yield strengths of the parent and weld metal are 543 MPa and 532 MPa, respectively, see Section 3.3.2) was observed at 3 mm below the weld top surface along the weld centerline for the as-welded specimen; see Figure 5a. High magnitude of tensile residual stress (in longitudinal direction) in this region may be due to the deposition of the last and final weld pass, and therefore, there is no further weld passes that could impart a tempering effect as it is the case for the previous passes in multipass welds. In addition, it is expected to have differential thermal gradient across the last weld pass as for every pass of multipass weld joint, but the lack of tempering effect in the last pass leaves the differential thermal gradient untouched exacerbating this effect (weld passes 20–25). However, with the increase in the depth of measurement (mid-plate thickness or 10 mm below the weld top surface) a reduction in the magnitude of residual stresses is detected due to the tempering effects of the subsequent passes; see Figure 5c. 

When the magnitude of the generated residual stress in this combined welding processes is compared with those of SMAW (investigated by the authors with the same methodology) [6], as the current preferred welding route for pipeline construction, it becomes clear that tensile residual stresses in MSAW+ FCAW combination are higher than SMAW (maximum residual stress for the combined MSAW+ FCAW and SMAW were 650 MPa and 506 MPa, respectively). Higher levels of tensile residual stresses (longitudinal direction) may be associated with the formation of more stressed phases such as Widmanstätten ferrite and bainite in the HAZ and WM of the MSAW + FCAW combination. High magnitude of residual stresses were significantly reduced after PWHT (Figure 5b,d) with a maximum stress of 144 MPa which is about 27% of the yield strength of the WM. Similar observation was made by Cho et al. [46] for various welding configurations (K-type and V-type weld joints) of SM400B steel where residual stresses were substantially decreased after PWHT (i.e., from 316 MPa to 39.3 MPa). 

Kernel Average Misorientation (KAM) parameter, which is calculated by EBSD, was used to estimate the residual strain distribution associated with non-uniform temperature distribution within the as-welded and PWHT specimens [47]. The main concept of assessing plastic strain via EBSD analysis is centered around this principle that the crystal lattice orientation within each grain becomes less homogenous with increasing plastic deformation [48]. EBSD is used to map the lattice orientations over a surface. The information is then used to calculate the differences in orientation, misorientation, between the lattice points. 

**Figure 6:**
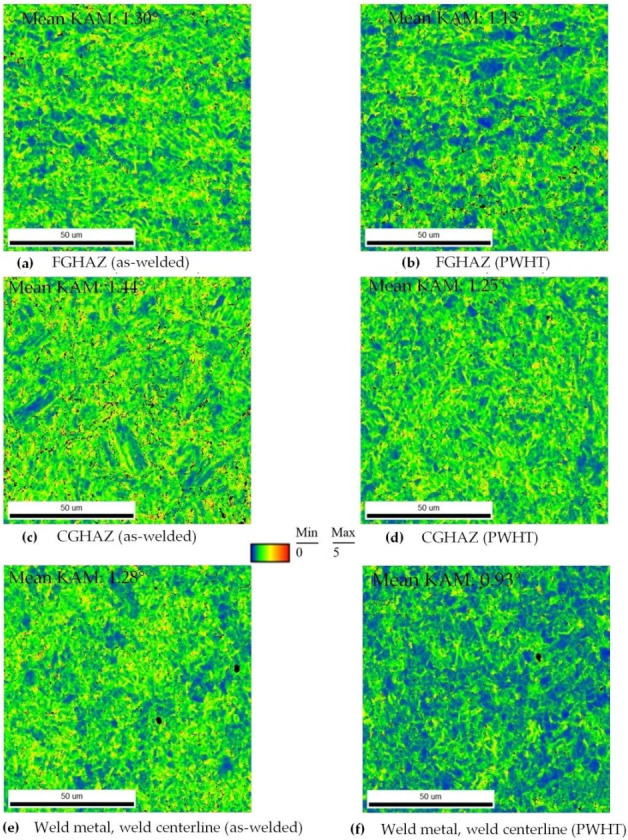
Superimposed Kernel Average Misorientation (KAM) maps for the Heat Affected Zone (HAZ) and Weld Metal (WM) regions of the as-welded (**a**,**c**,**e**) and post-weld heat treated samples (**b**,**d**,**f**).

The KAM mapping illustrates the mean angle between the crystallographic orientation of each pixel and other pixels at its nearest neighborhood (only misorientations ≤ 5° are considered for the KAM calculation to ensure any contribution from high angle grain boundaries is ignored). The second nearest neighbors were selected to define the kernel in this investigation. Figure 6 shows the KAM maps with the mean KAM values for the FGHAZ, CGHAZ, and weld center regions of the as-welded and post-weld heat treated samples. It is evident that the degree of plastic deformation, and thus the residual strain, present in the microstructure (FGHAZ, CGHAZ, and WM) decreases after the PWHT (it should be noted that the blue color represents the region with minimal plastic deformation and the red color is showing the region with the highest plastic deformation). 

### 3.2. Microstructure Analysis

**Figure 7:**
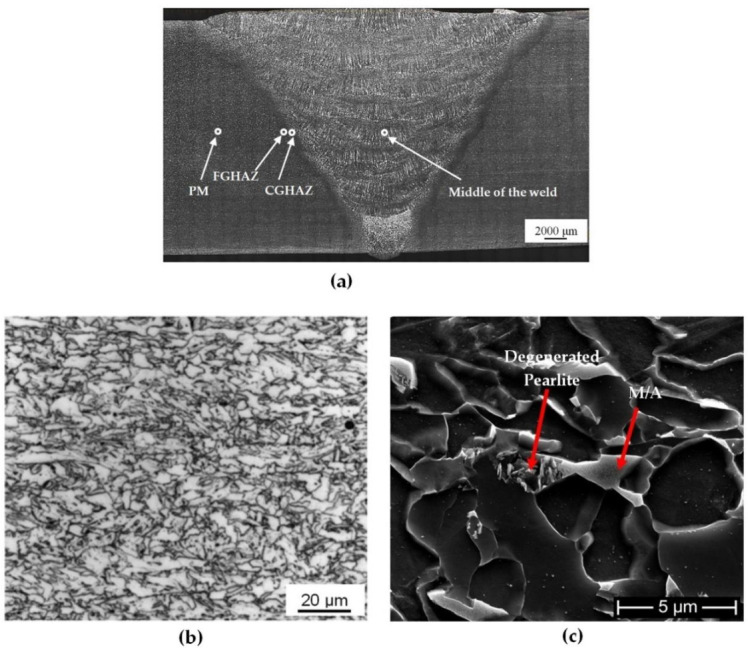
(**a**) Optical macrograph of weld joint showing the location of micrographs taken for the PM and WM. (**b**) Optical micrograph of the the as-received X70 steel (PM) showing mainly granular bainite (GB) and polygonal ferrite with some Martensite–Austenite (M–A)/degenerate pearlite mixture, (**c**) FEG-SEM micrograph of dark area in (**b**) [51].

The optical macrograph shown in Figure 7a clearly shows the formation of about 1–2 mm thick HAZ, the MSAW weld metal (first pass), and the remaining FCAW multipass weld joint along with the PM. The X70 PM has a microstructure primarily consisting of granular bainite with some instances of polygonal ferrite along with some dark color regions that may resemble pearlitic structure as shown in Figure 7b. When observing the PM at high magnification under SEM, the dark color regions appear to constitute of M–A constituents and degenerate pearlite (Figure 7c). M–A constituents are formed because of austenite stabilizing elements, such as manganese and nickel, that allow austenite to exist at room temperature [49]. Note in Table 1, the as-received X70 PM contains some nickel, a strong austenite stabilizer [50].

**Figure 8:**
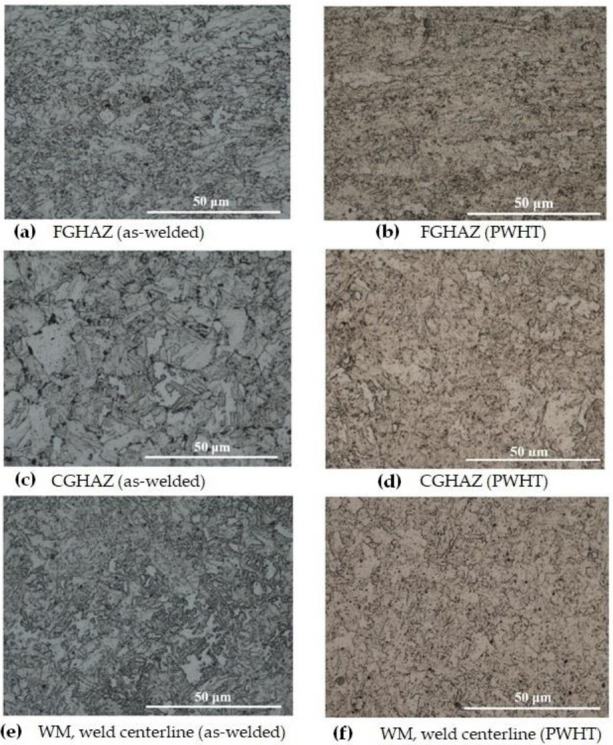
Optical micrographs showing the FGHAZ, CGHAZ, and the WM in the as–welded (**a**,**c**,**e**) and after PWHT (**b**,**d**,**f**) specimens.

**Figure 9:**
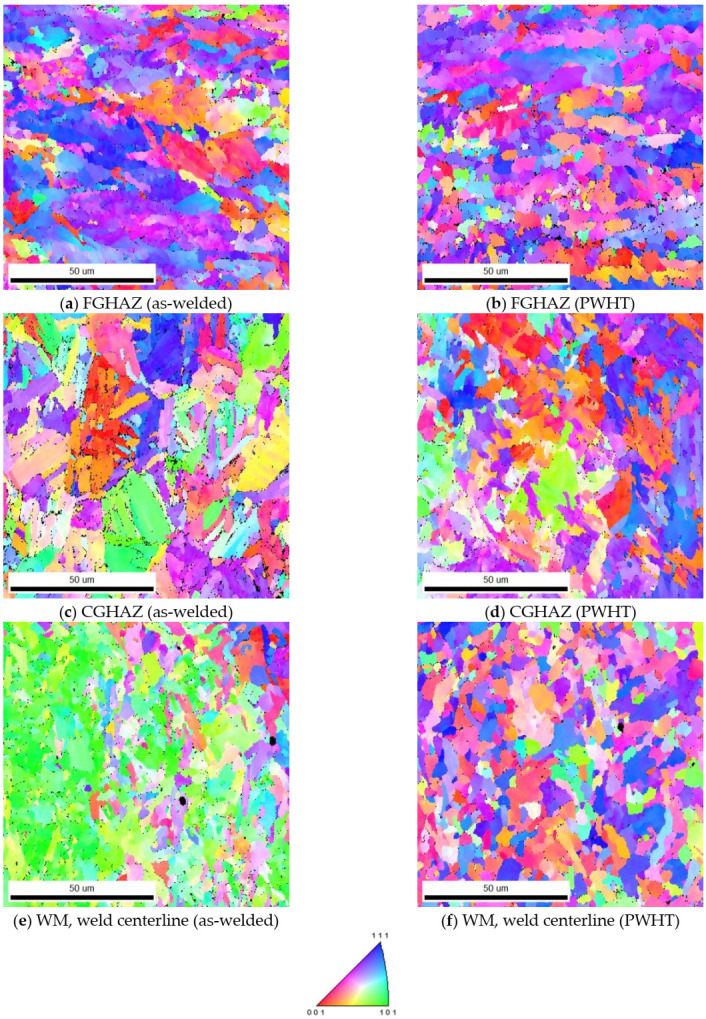
EBSD IPF (Inverse Pole Figure) micrographs showing the FGHAZ, CGHAZ and the WM in the as-welded (**a**,**c**,**e**) and after PWHT (**b**,**d**,**f**) specimens.

The optical and EBSD micrographs of Figure 8 and Figure 9, taken from the HAZ and weld regions specified on the weld joint optical macrograph, Figure 7a, illustrate the typical macrostructures of the HAZ regions (CGHAZ and FGHAZ) and the WM (weld centerline), for both as-welded and PWHT specimens. There is a continuous grain growth detected in the HAZ regions, for the as-welded specimen, toward the fusion zone with the microstructure being predominately acicular ferrite in the FGHAZ (Figure 8a), changing to mainly bainitic structure (Figure 8c), as it approaches fusion zone (CGHAZ). However, there are other morphologies of Widmanstätten and polygonal ferrite also detectable, but the dominant phases are acicular ferrite and bainite for FGHAZ and CGHAZ respectively. The WM, however, is predominantly acicular ferrite with some polygonal and Widmanstätten ferrite morphologies (Figure 8e). There is, however, changes taking place within different ferritic phases during the course of PWHT process, where more stable phase of polygonal ferrite forms. These changes are also evident in the WM, where for the as-welded specimen finer ferritic microstructure (Widmanstätten, bainite, polygonal and acicular ferrite) are transformed to mainly coarsened equiaxed polygonal ferrite. More homogeneous microstructure (i.e., grain size) was also observed across the weld/HAZ of the PWHT sample, whilst the slightly larger grains are expected.

Previous investigation conducted by Filho et al. [29] draws similar conclusions where applying normalizing heat treatment for low alloy steel weld metals led to microstructural changes in which fine grained microstructure was changed to a coarse equiaxed ferrite with ferrite-carbide aggregates. 

The application of EBSD enables to better differentiate between the morphologies of the weld micro-constituents as illustrated in Figure 9. The formation of a mainly acicular ferrite morphology for the FGHAZ (Figure 9a) and WM (Figure 9e) and formation of mainly bainitic morphology for CGHAZ (Figure 9c) are easily recognizable. Furthermore, the formation of coarsened structures for FGHAZ and CGHAZ and transformation of acicular ferrite into polygonal ferrite for the WM in the PWHT samples are also well-represented in Figure 8b,d,f. EBSD analysis also provides information on the crystallographic orientations of constituent phases and imperfections such as grain boundaries enabling to distinguish between low and high angle boundaries through degrees of misorientations. It further provides a useful visualization of the microstructure. 

**Figure 10:**
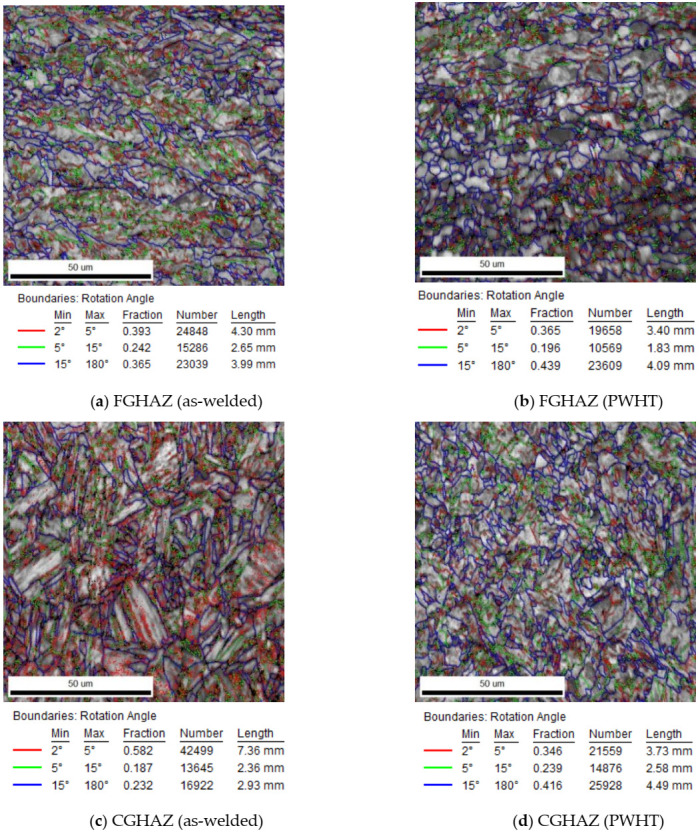

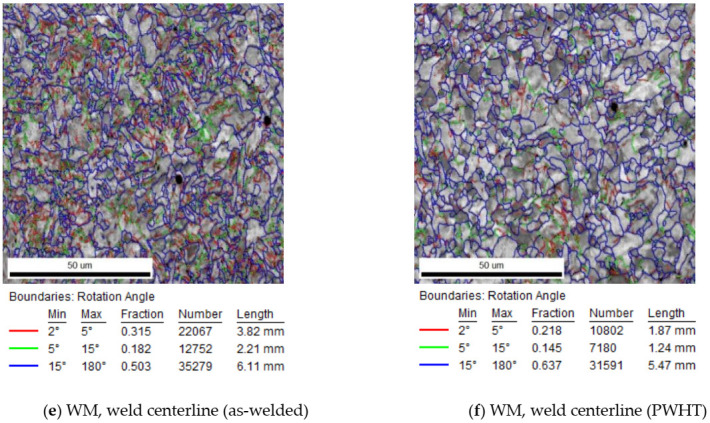
Misorientation maps of the as-welded (**a**,**c**,**e**) and PWHT sample (**b**,**d**,**f**) showing high angle boundaries for PWHT specimen (≥15°).

**Figure 11:**
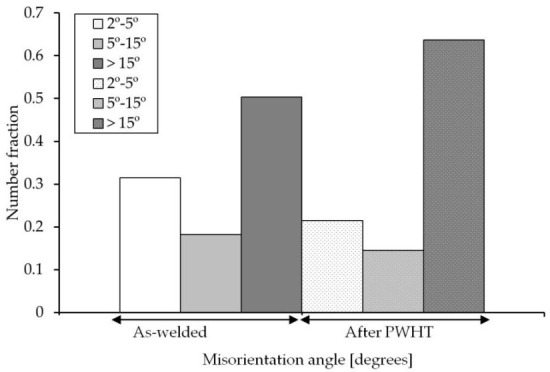
Misorientation angle distributions for the WM (as–welded and PWHT specimen), taken from weld centerline in the middle of plate thickness.

The Image Quality (IQ) maps presented in Figure 10a–d show the morphology of ferritic phases formed within the HAZ either close to the PM (Figure 10a,b) or close to the weld fusion zone (Figure 10c,d). In confirmation of observations in Figure 8, the IQ image of the HAZ near the PM (FGHAZ) is mainly consisted of acicular ferrite while the HAZ near fusion zone (CGHAZ) is mainly bainitic for the as-welded specimen. Both FGHAZ and CGHAZ regions have a large proportion of boundaries with low angle misorientations <15° while in the case of PWHT, there are mainly polygonal ferrite comprising a large proportion of boundaries with misorientations angles >15°. The quantitative analysis of the grain boundary misorientation of the WM is shown by the distribution histogram in Figure 11 for both as-weld and PWHT specimens (weld centerline in the middle of plate thickness). The WM for the as-welded specimen has a large proportion of boundaries with low angle misorientations <15°, while in the same location for the PWHT (see Figure 11), consists of a large proportion of boundaries with high angle misorientations >15°. A reduction in low angle boundary or an increase in high angle boundaries are indications of phases with mainly low angle boundaries (i.e., bainite with low angle lath boundary) are changing to polygonal ferrite with high angle boundaries. The low to intermediate misorientation angle populations, particularly for the as-welded specimen, may be the misorientation angles initiated from the prior austenite grain boundaries [52,53].

The effective grain size was measured by the linear intercept method from the EBSD analysis, average of 10 lines, with a misorientation angle > 15° criterion. It was found that the grain size is larger in the PHWT sample as compared with the as-welded one in the FGHAZ, the CGHAZ and the WM, as shown in Table 2.

**Figure 12:**
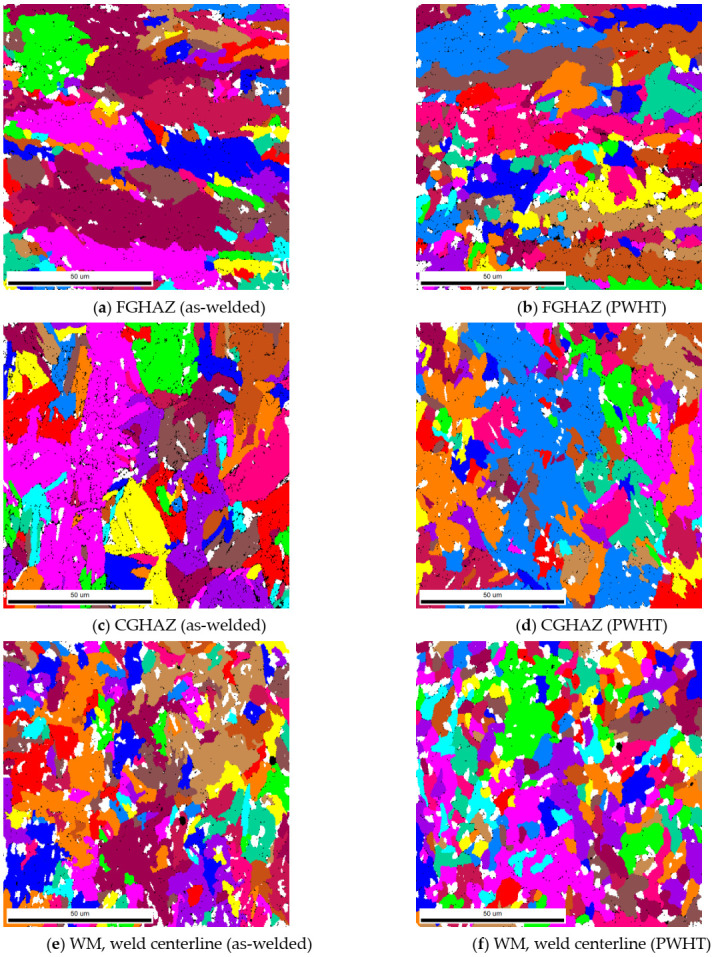
EBSD grain maps micrographs showing the FGHAZ, CGHAZ and the WM in the as-welded (**a**,**c**,**e**) and after PWHT (**b**,**d**,**f**) specimens.

As shown in the Table 2, the CGHAZ is smaller than the FGHAZ. This may be attributed to sectioning of the metallographic samples as shown in the grain maps in Figure 12. The large pink grain (Figure 12c) is basically the representative of grain structure, which is clearly larger than the grains in FGHAZ region. However, it is seen that certain grains were sectioned in such a way that the revealed grain size appeared to be smaller than the grain seen in FGHAZ.

**Figure 13:**
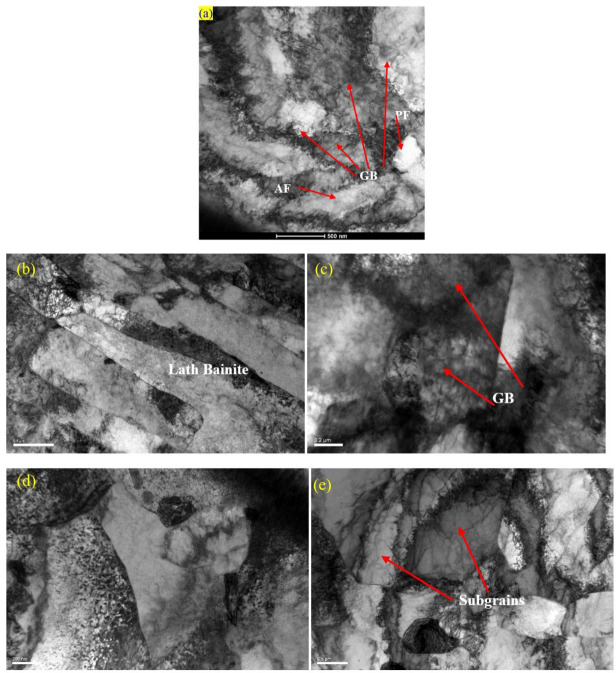
TEM micrographs to show the microstructural changes during welding and after PWHT for; (**a**) PM, (**b**) HAZ (as weld), (**c**) WM (as weld), (**d**) HAZ (PWHT), and (**e**) WM (PWHT sample). (**a**) PM-acicular ferrite (AF), granular bainite (GB) and polygonal ferrite (PF); (**b**,**c**) As weld: TEM micrographs to confirm the formation of highly dislocated structure of as-weld including the formation of lath bainitic region in HAZ; (**d**,**e**) PWHT: TEM micrographs to show the process of polygonization and the formation of subgrains as well as the formation of polygonal ferrites.

The formation of different morphologies of phases was further confirmed by TEM analysis, especially the microstructural changes due to PWHT. As shown in Figure 13, the as-received PM comprises mainly a bainitic structure while the weld structure shows a highly dislocated structure. Upon PWHT, the rearrangement of dislocations and the formation of subgrains are well depicted in the TEM micrograph which is expected to have a reduced residual stress in confirmation of neutron diffraction results in Figure 5 and EBSD analysis of Figure 6. Study conducted by Zhang et al. [54] also confirmed softening of HAZ and microstructural changes in the CGHAZ in which quenched martensite of China Low Activation Martensitic (CLAM) steel showed a tendency of polygonization after PWHT.

### 3.3. Mechanical Properties of the Weld

#### 3.3.1. Hardness

**Figure 14:**
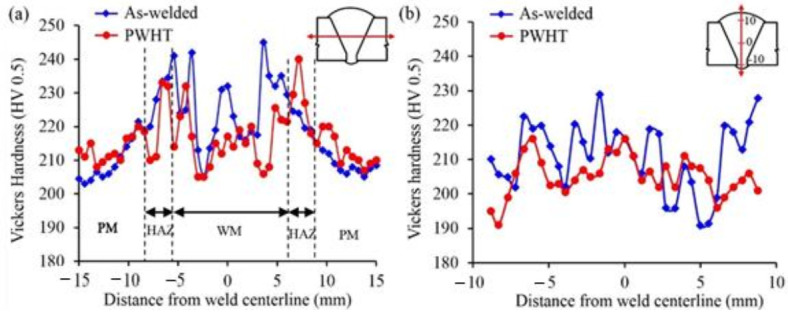
Microhardness distribution for the as–welded and PWHT specimens; (**a**) mid thickness across the weld, (**b**) through thickness.

Figure 14 shows the microhardness distributions across the as-welded and PWHT joints. The difference in microhardness is believed to be due to the differences in the morphology and distribution of microconstituents of the as-welded and after PWHT samples including the level of dislocation density of the as-weld specimens. The formation of acicular and bainitic ferrite along with finer grain size and higher dislocation density are responsible for high hardness values in the WM and HAZ of the as-welded specimens. The PWHT microstructure comprising mainly polygonal ferrite having a rearranged dislocation population in the form of subgrain boundaries (lower dislocation density) and larger grain size which are responsible for lower hardness values for PWHT samples. This finding is in-line with the Hall-Petch equation where hardness is dependent on the reciprocal square root of the grain size ($H=H_{0}+K_{H}d^{-1/2}$) [55], plus the fact that similar trend was reported in previous studies [24,56,57]. In the Hall-Petch equation, *H* is the hardness (HV), *d* is the grain size, *K_H_* is the Hall-Petch slope and *H_o_* is the hardness intercept at *d*^−1/2^ = 0.

Furthermore, the value of transverse hardness in the PM appears to have not been affected by the PWHT and hardness values are in the range of 204–214 HV0.5 (Figure 13a). The through-thickness hardness value, however, is slightly higher for the as-welded specimen, specifically for the last weld passes with the minimal tempering effects and higher cooling rates (weld passes 20–25). The PWHT provides the tempering treatment absent in the as-welded specimen and thus the hardness dropped slightly to around 198 HV0.5.

#### 3.3.2. Tensile Test

**Figure 15:**
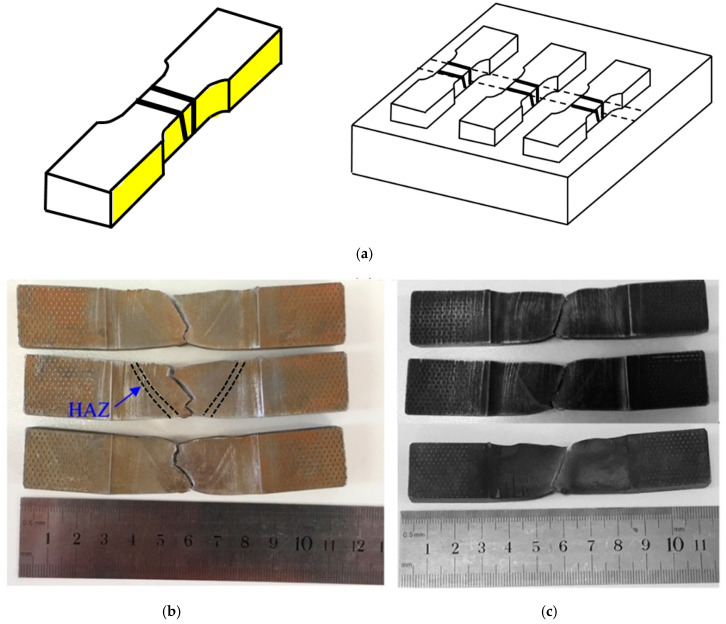
Tensile test specimens of X70 weldments both in the: (**a**) side view of the fractured specimen (**b**) as-welded and (**c**) PWHT conditions showing the fracture at the fusion zone (sub-sized tensile specimens).

**Figure 16:**
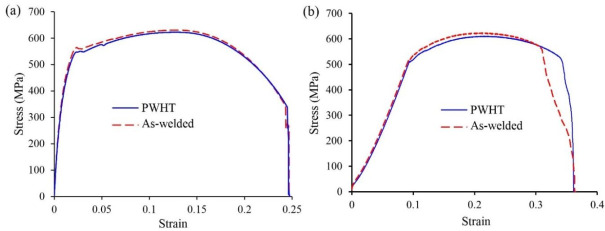
Engineering stress-strain curves for both specimens in; (**a**) standard size specimen, and (**b**) sub-sized specimen.

The tensile tests were initially conducted for the standard size specimens and the crack initiation and propagation were in the PM for all of the samples, further away from the WM and HAZ. Therefore, it was decided to conduct tensile tests with the sub-sized specimens to fully characterize mechanical properties in the WM and HAZ of the welded samples. Figure 15a,b shows the fractured tensile specimens for the as-welded and PWHT coupons (sub-sized tensile specimens). As seen, in both cases the cracks initiated within the WM close to the HAZ and propagated through the thickness at a nearly 45° with respect to tensile axis. The crack initiation in the as-welded specimens could be attributed to the formation of high level of residual stresses in this region during welding (as shown in Figure 5) and/or formation of geometric stress raisers such as wagon track (elongated slag inclusions) or undercut (groove which occurs near the toe of weld) [58]. The crack propagation was with higher rate for the as-welded coupons with the development of cracks from the top surface as well as near the weld root simultaneously, as shown in Figure 15a. Such behavior might be related with the release of high magnitude of tensile residual stresses in the as-welded specimen. This phenomenon reduced the elongation of the as-welded specimens, as shown in Figure 16b, in comparison with the PWHT specimen. Similar finding was reported in the study conducted by Zhao et al. [59] where utilizing PWHT lead to improvement of the ductility of the heat treated specimens at the expense of strength. For PWHT specimens, the crack always initiated at the weld top surface and propagated towards the weld root at nearly 45 degrees with respect to the tensile axis. As discussed earlier, for the standard size tensile specimens (Figure 3b), the fracture was occurred in the PM far away from the WM and HAZ.

Moreover, the fracture surface in all cases shows ductile fracture characteristics with dimpled fracture surface). Figure 16a,b are typical tensile diagrams for the standard and sub-sized specimens respectively. For the as-welded sample (sub-sized), there is some anomaly at the end of tensile test where the stress-strain curve shows some further elongation at fracture. This may be explained with respect to the crack path seen in the middle as-welded sample in Figure 15a. The crack propagation from both sides ends up in the middle with some step characteristics. The extra elongation may be due to joining of the two crack paths from either side of the sample.

Table 3 also shows the tensile properties for the as-welded and PWHT specimens. A slight decrease in the yield (the 0.2% yield strength for the as-welded and after PWHT were 532 MPa and 513 MPa, respectively) and tensile strengths of post-weld heat treated specimens can be seen which may be attributed to the stress-relieving as well as higher grain size (Table 2) and phase transformations (changing the bainitic, Widmanstätten and acicular ferrite to mainly polygonal ferrite) in the samples. It must be noted that the reduction of yield strength of the post-weld heat treated specimen is compatible with Hall-Petch equation, where higher grain size for heat treated specimen is resulted in lower yield strength. Furthermore, higher ductility was observed after heat treatment process where the percentage of elongation for the as-welded and PWHT specimens were 29% and 33%, respectively which could be due to stress-relieving due to PWHT. Similar findings were reported elsewhere where utilizing PWHT resulted in higher ductility for the quenched and tempered (RQT) high strength steel weld (grade S690) and P91 steel with an increase of 14.16% for V-groove weld as reported by Zhao et al. [59] and Pandey and Mahapatra [60], respectively.

**Figure 17:**
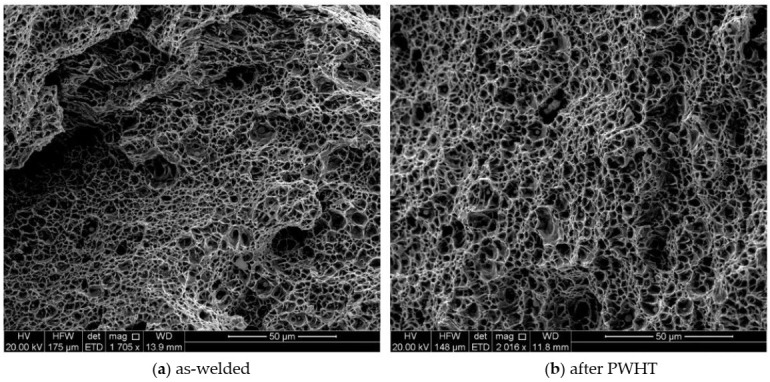
SEM fractographs of tensile specimens for (**a**) as-welded and (**b**) after PWHT.

High proportion of high-angle grain boundaries, particularly for the PWHT specimen (Figure 10 and Figure 11) act as an obstacle to cleavage crack propagation [61] leaving a mainly dimpled fracture surface. The dimples evident in Figure 17 are indicative of a ductile mode of fracture. It is also noticeable that the sizes of dimples are finer for the as-weld specimen which is an indication of lesser void coalescences with lower ductility. The dimples observed in the SEM micrographs in Figure 17 appear to have originated at non-metallic inclusions within the WM, if the micrographs are examined closely. During ductile fracture of the WM, the energy required for interface decohesion at the inclusions is generally low due to incoherent interface at the inclusions and crack propagates by the growth and eventual coalescence of such microvoids [62].

#### 3.3.3. Impact Test

**Figure 18:**
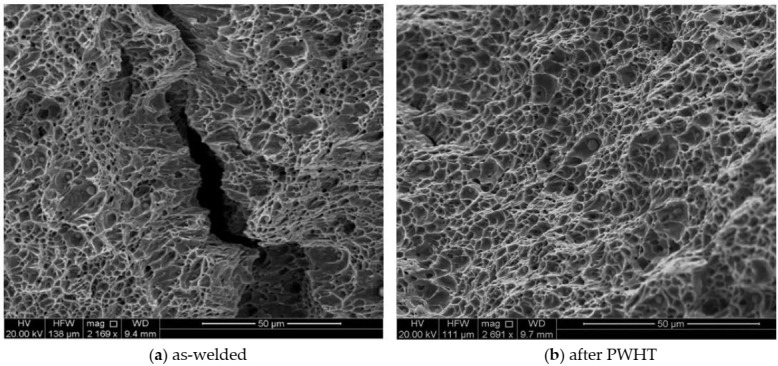
SEM fractographs of impact specimens for (**a**) as-welded and (**b**) after PWHT.

The Charpy impact test was performed to evaluate the toughness (J) of welded joints for both specimens (as-welded and after PWHT). The impact specimens were prepared in three different locations transverse to the weld direction (V-notch was positioned in the weld centerline), as shown in Figure 4. The average toughness for as-welded and PWHT specimens was found to be 128.5 J and 126.3 J, respectively. Figure 18a,b displays the fractographs of notched impact specimens for the as-welded and after PWHT joints, respectively. The displayed fractographs consist of small and uniform dimples, which are an indication that the specimens failed in a ductile manner under the action of impact loading. Small inclusions inside the dimples were observed for both PWHT and as-welded specimens. It seems that the size of dimples is slightly smaller for the as-welded sample confirming the small differences in the toughness values.

## 4. Conclusions

In this investigation, the effect of PWHT on the residual stresses, microstructural changes, and mechanical properties of a multi-pass HSLA weld joints made by a combined MSAW and FCAW processes were studied. The following conclusions could be drawn from the present study:

1. The combined MSAW-FCAW welding of X70 pipeline steel results in higher residual stresses when compared to the work done by the authors on SMAW [6] but renders a faster fabrication with as-good if not better weld joint quality as SMAW.
2. PWHT leads to microstructural changes including polygonization, rearrangement of dislocations and the formation of subgrains which is in line with significant reduction of residual stress in the heat-treated specimen.
3. The acicular ferrite and granular bainite formed in the as-welded specimen (HAZ and WM) were transformed into mainly polygonal ferrite. Moreover, a more homogeneous microstructure in terms of grain size was found after the PWHT process (HAZ and WM).
4. Microstructural changes which occur during PWHT (i.e., subgrain formation and grain boundary misorientations) explains the mechanical behavior with the increase in the elongations (higher ductility) and a slight reduction in yield strength of the PWHT specimen.
5. There was a minor decrease in the impact toughness of the welded joints after PWHT, less than 2% reduction. Moreover, there was no noticeable change in the hardness of the base material but PWHT provides the tempering treatment absent in the as-welded specimen, particularly for the upper layer of the weld, and thus the hardness dropped from the average of 221 HV0.5 (as-welded sample) to 198 HV0.5 in these regions. Some level of softening was also observed in the WM after PWHT.
6. High value of the tensile residual stresses, present in the as-welded specimen, reduced substantially after PWHT. It is about 27% and 20% of the yield strength of the WM in the longitudinal and transverse directions, respectively.

## Figures and Tables

**Table 1:** Chemical composition of filler materials and PM (wt.%)**.**

Chemical Composition	ER 70S-6 [35]	E81T1-Ni 1M [35]	PM [36]
%C	0.09	0.04–0.05	0.059
%Mn	<1.60	1.26–1.36	1.57
%S	0.007	0.006–0.009	<0.002
%Si	0.90	0.25–0.29	0.19
%P	0.007	0.005–0.008	0.011
%Cu	0.20	-	0.16
%Cr	0.05	0.04–0.05	0.032
%Ni	0.05	0.86–0.96	0.19
%Mo	0.05	0.01	0.17
%V	0.05	0.02–0.03	0.027
%Ti	–	–	0.01
%NB	–	–	0.045
%N	–	–	0.004

**Table 2:** Grain size measurement by linear intercept method with random test lines (average of 10 lines) on the EBSD scans (misorientation angle > 15° criterion).

Sub-Zone	As-Welded (µm)	PWHT (µm)
FGHAZ	3.01	2.62
CGHAZ	2.04	2.24
Weld Middle	1.84	2.14

**Table 3:** Tensile properties for the as-welded and PWHT specimens.

X70 Steel	0.2% Yield Strength (MPa)	Ultimate Tensile Strength (MPa)	Percentage of Elongation (%)
PM	543 ± 4.7	623 ± 6.9	24.0 ± 0.2
As-welded (WM specimens)	532 ± 5.6	618 ± 6.3	28.5 ± 0.3
PWHT (WM specimens)	513 ± 4.6	607 ± 4.2	33 ± 0.4
